# Facilitators and barriers to HIV pre-exposure prophylaxis (PrEP) uptake through a community-based intervention strategy among adolescent girls and young women in Seme Sub-County, Kisumu, Kenya

**DOI:** 10.1186/s12889-021-11335-1

**Published:** 2021-07-01

**Authors:** Maya Jackson-Gibson, Ashley Uzoamaka Ezema, Wicklife Orero, Irene Were, Ramael Osasogie Ohiomoba, Patrick Owuor Mbullo, Lisa Ruth Hirschhorn

**Affiliations:** 1 Northwestern University Feinberg School of Medicine, Chicago, Illinois USA; 2 Pamoja Community Based Organization, Kisumu, Kenya; 3 Department of Anthropology & Global Health, Northwestern University, Chicago, Illinois USA; 4 Department of Medical Social Sciences, Northwestern University Feinberg School of Medicine, Chicago, Illinois USA

**Keywords:** Adolescent girls and young women, DREAMS initiative, HIV pre-exposure prophylaxis, Consolidated framework for implementation research, Kenya

## Abstract

**Background:**

While the introduction of HIV Pre-Exposure Prophylaxis (PrEP) as an HIV prevention strategy has allowed women to exercise more control over the reduction of HIV transmission rates, adolescent girls and young women in Sub-Saharan Africa continue to experience higher rates of HIV infections and bear the greatest disease burden. Understanding progress in PrEP uptake among adolescent girls and young women would enhance risk reduction in this vulnerable population. The Determined, Resilient, AIDS-Free, Mentored and Safe women (DREAMS) Initiative plays a key role in this risk reduction strategy.

**Methods:**

We performed a qualitative study to explore facilitators and barriers to PrEP implementation and assess factors effecting initiation and persistence on PrEP among adolescent girls and young women enrolled in the DREAMS Initiative at Pamoja Community Based Organization in Kisumu, Kenya. We conducted key informant interviews (*n* = 15) with Pamoja Community Based Organization staff, health care providers and community leaders. Additionally, we conducted focus group discussions with young women receiving PrEP and peer mentors (*n* = 40). We performed a directed content analysis using the Consolidated Framework for Implementation Research to organize the identified facilitators and barriers.

**Results:**

We found that the use of the safe space model, decentralization of PrEP support and delivery, peer mentors, effective linkage to local health care facilities, the sensitization of parents and male sexual partners, disclosure of PrEP use by beneficiaries, active stakeholder involvement and community engagement were among some of the facilitators to PrEP uptake. Barriers to PrEP implementation, initiation and persistence included stigma associated with the use of anti-retroviral drugs, drug side effects, frequent relocation of beneficiaries, limited resources for routine screening and medication monitoring, and a limited number of qualified health care workers for PrEP distribution and administration.

**Conclusion:**

Overall, the community roll-out of PrEP within the DREAMS Initiative was successful due to a number of key facilitating factors, which ultimately led to successful PrEP implementation, increased PrEP initiation and enhanced persistence among adolescent girls and young women. The identified barriers should be addressed so that a larger scale-up of PrEP roll-out is possible in the future.

## Introduction

Despite global advances in HIV prevention and treatment, Sub-Saharan Africa still bears the greatest disease burden with over 50% of people living with HIV, with women being the most disproportionately impacted. This high burden is due to a combination biological, social, cultural, behavioral, economic and structural factors [1, 2]. In 2018, the UNAIDS Analysis of HIV Prevalence in Kenya reported that women accounted for 65% of people living with HIV in this region, with an incidence more than double among young women (11,000) than young men (5000) [3]. Several interventions, including behavior change and condoms, have been applied to help reduce the spread of HIV, especially among adolescent girls and young women. These interventions have been shown to be limited in their ability to promote HIV risk reduction because they are dependent on male partner cooperation [4]. HIV Pre-Exposure Prophylaxis (PrEP) has been proven to reduce the transmission of HIV infections for individuals who are at high risk [5]. Unlike other methods, PrEP represents an effective biomedical HIV prevention method, with the potential of giving a woman more self-efficacy and agency by taking a once-daily pill to minimize her HIV risk.

The Determined, Resilient, Empowered, AIDS free, Mentored, and Safe women (DREAMS) Initiative, was designed to reduce the burden of HIV and early pregnancies on adolescent girls and young women between 10 to 24 years of age in Sub-Saharan Africa by 40% in two years [6–8]. Funded by the United States Presidential Emergency Plan for AIDS Relief (PEPFAR) and private sector partners, DREAMS consists of biological, structural, and behavioral interventions to address risk factors that influence HIV transmission among adolescent girls and young women. Some of these risks include sub-optimal healthcare access, gender inequality, gender-based violence, poverty, and poor education [2]. Combined, these three interventions have shown the potential to significantly reduce HIV incidence amongst adolescent girls and young women. These interventions achieve success through the creation of social assets, promotion of health services, strengthening of family and male sexual partner engagement in HIV risk reduction programming, and reduction of stigma associated with PrEP use within the greater community [8, 9].

PrEP is one of core intervention of the DREAMS Initiative. Others include post-violence care, mixed contraceptive methods, and HIV testing services. Despite the efficacy in clinical trials, the uptake of PrEP by this population in Sub-Saharan Africa has been slow. Known barriers include a) consistent access to PrEP, b) capacity of community-based organizations to roll-out the DREAMS Initiative and PrEP services, c) limited number of health care providers trained to provide PrEP, d) community stigma against PrEP use, and e) individual knowledge and beliefs surrounding PrEP [4, 10]. Known facilitators to PrEP implementation include a) the engagement of community stakeholders, b) the use of safe spaces and peer mentors c) stigma reduction through continuous sensitization d) and disclosure of PrEP use by beneficiaries [4, 10]. In areas where the DREAMS Initiative has been implemented, efforts to overcome barriers faced by PrEP implementation strategies, such as PrEP initiation and persistence have shown some success [11]. These gains are attributed to understanding the needs of adolescent girls and young women in the context of their local community, increased emphasis on adolescent girl and young women empowerment, and addressing structural barriers.

Initiatives led by community-based organizations (CBOs) can play a significant role in mitigating these barriers to PrEP initiation and persistence by addressing the socioeconomic and health systems barriers while supporting continuous education, awareness campaigns, and distribution of PrEP. In addition, these grassroots organizations can be well equipped to address community and individual challenges to acceptance and persistence [12]. Pamoja Community Based Organization (referred to as Pamoja) is a grassroot, non-profit organization based in Kisumu County, Kenya. In partnering with the Kenya Ministry of Health (MoH), it became a primary implementer of the DREAMS Initiative within the Seme Sub-County of Kisumu County, one of the areas prioritized for PrEP roll-out by the national government due to the high disease burden in this region [13]. Kisumu County continues to have a high prevalence rate of HIV despite a declining national rate. This region has many overlapping risk factors, leaving adolescent girls and young women more vulnerable to newly acquired HIV infections. These risk factors include social (boda boda drivers, early sexual debut), cultural (wife inheritance/widows, multiple sexual partners) and economic (unemployment, poverty) factors [14].

The Kenya Ministry of Health formed a partnership with Pamoja to implement PrEP through the DREAMS Initiative. The implementation followed the Kenya Ministry of Health PrEP Implementation Framework of 2017 and the National Antiretroviral Therapy (ART) guidelines from 2018. PrEP initiation is done at Kenya MoH-supported facilities, with ongoing prescribing done at those facilities or through MoH health care workers in the safe spaces. The Kenya MoH has been able to ensure a consistent and adequate supply of PrEP by partnering with the Kenya Medical Supplies Agency. In addition to PrEP initiation and drug stock outs, the Kenya MoH is also responsible for data quality monitoring.

The goal of our qualitative study was to further explore some of the known facilitators and barriers to PrEP implementation, uptake and persistence among adolescent girls and young women within the DREAMS Initiative at Pamoja using a directed content analysis [15]. We used the Consolidation Framework for Implementation Research (CFIR) as our organizational framework. CFIR is an implementation research framework designed to analyze qualitative data and to identify facilitators and barriers influencing implementation outcomes [16]. These known facilitators and barriers formed the basis of our interviews, while we also explored additional themes that emerged from the qualitative interviews. Our findings can help inform similar and new efforts to implement PrEP and increase PrEP uptake and persistence among adolescent girls and young women in Kenya and other similar settings.

## Methods

### Study design

We conducted a qualitative study to identify and understand the individual, community and program-level factors which facilitated or hindered PrEP implementation, PrEP initiation and persistence among the adolescent girls and young women participating in the DREAMS Initiative delivered by Pamoja in Seme, Kisumu County. We considered PrEP integration into the DREAMS Initiative as the intervention and the main focus of our study.

### Study setting

The study was conducted at Pamoja in East Seme Ward, Seme Sub-County, in Kisumu County. This area is predominantly Luo by ethnicity and is ranked one of the poorest sub-counties in Kisumu with over 80% of the population unemployed, and 40% living below the poverty line [17]. Pamoja has enrolled 4,831 adolescent girls and young women in its DREAMS program over the last four years. Since 2017, 938 young women have been initiated on PrEP. Pamoja implements PrEP by engaging community and health facility platforms while integrating it into the overall DREAMS empowerment and education package. At the community level, Pamoja organizes meetings with local chiefs, assistant chiefs, and community members at communal centers to educate them on the benefits of PrEP for adolescent girls and young women, combat medication associated stigma, and gain more support for the PrEP program. Additionally, Pamoja conducts sensitization meetings for adolescent girls and young women enrolled in the DREAMS program perceived to be at risk for HIV transmission. These meetings occur at designated safe spaces (girls-only meeting places) where adolescent girls and young women meet and have discussions facilitated by trained peer mentors regarding issues affecting their well-being [18]. These meetings are overseen by expert nurses or clinical officers from government health facilities.

When an adolescent girl or young woman qualifies for PrEP, she is initiated on the medication at the clinic by a health care provider working in a government clinic. Subsequent follow-up appointments and refills take place at the community safe space. Adolescent girls and young women who are newly initiated on PrEP are given monthly clinic appointments to closely monitor medication persistence and side effects. These clinic appointments are then scheduled after every 2–3 months if the PrEP beneficiary maintains medication persistence without any reported side effects. We defined ‘PrEP initiation’ as an adolescent girl or young woman starting PrEP through Pamoja, and ‘medication persistence’ as her ability remain explicitly on PrEP and take the medication as prescribed [19].

### Participants

We employed purposeful sampling to ensure representation from the five catchment areas that are the main focus for Pamoja outreach and community-based initiatives. Within each area, we did convenience sampling to recruit peer mentors, Pamoja staff, health care providers, and local chiefs and assistant chiefs. Peer mentors, Pamoja staff, and healthcare providers were eligible if they were actively involved in the PrEP program. This included recruitment, managing of safe spaces, community sensitization, or the prescribing and follow up of PrEP. One chief or assistant chief was selected from each of the five catchment areas. DREAMS beneficiaries eligible for this study needed to be between 16 to 24 years of age and actively taking PrEP (young women 16 years of age are considered “mature minors” in Kenya). Using a list of beneficiaries initiated on PrEP generated from the DREAMS database maintained by Pamoja, we sampled from each catchment area to ensure equal representation of adolescent girls and young women from all areas. Beneficiaries younger than 15 years of age or younger could not be included in our study due to Institutional Review Board restrictions. DREAMS beneficiaries not actively taking PrEP or unable or unwilling to give consent were excluded from our study. Eligible participants were approached by Pamoja staff and asked to participate in the study. Written consent was obtained from all participating individuals.

### Data collection

We developed semi-structured interview guides for key informant interviews (KIIs) and focus group discussions (FGDs) focusing on the process of PrEP integration into the DREAMS program adopted by Pamoja, the knowledge and attitudes of adolescent girls and young women and key informants around PrEP, and the facilitators and barriers to medication initiation and persistence. The interview guides were developed by members of the research team and were based on our literature review and related field experience of the senior authors [LRH and POM]. We conducted five FGDs with eight participants, three with PrEP recipients and two with peer mentors (total *n* = 40). We performed 15 KIIs including Pamoja staff (*n* = 5), health care providers (*n* = 5), and local chiefs and assistant chiefs (*n* = 5). Interviewers included one research staff member from Pamoja who could speak English and the local language (Luo) and the first author (MJG). Both interviewers were trained in conducting qualitative interviews. They conducted interviews in either the local language or English based on the preferences of the participant. The number of FGD and KIIs was limited by available resources and time.

### Data analysis

Data were transcribed, translated into English if necessary. We generated an a priori coding structure based on key concepts identified by other researchers in prior studies and coded by one author (MJG) using Atlas.ti, version 8.4.4 (1135) software. Based on the constant comparative method, additional codes were incorporated into this coding scheme as new themes emerged from the interviews [20]. A co-author (AE) coded a subset of three interviews in parallel. The differences in coding were discussed until a shared understanding was reached. A third author (POM) reviewed all coding to ensure consistency. Fifteen codes remained after coding was completed. Using directed content analysis, and CFIR as the organizing structure, we grouped the identified patterns of facilitators and barriers into relevant domains and constructs of that framework [15, 16, 20, 21]. Definitions of the specific domains and constructs containing the barriers and facilitators are displayed in Table 1. Final quotations which were representative of the specific theme were chosen for inclusion in the manuscript.

**Table 1:** Summary of main themes based on CFIR domains and associated constructs

Definitions of CFIR domains used and associated construct definitions (CFIRguide.org) which emerged as main themes	Facilitators	Barriers
**Inner Setting:** Characteristics of the implementing organization such as team culture, relative priority of the intervention, leadership engagement, and the compatibility of the intervention with the organization		–
***Compatibility:*** “The degree of tangible fit between meaning and values attached to the intervention and how the intervention fits in with the existing goals, workflows, and systems of the organization.”	Pamoja staff believed that the implementation of PrEP through the DREAMS Initiative was critical in achieving the goal of reducing the amount of new HIV amongst adolescent girls and young women in their community.	–
***Relative Priority:*** “Individuals’ shared perception of the importance of the implementation within the organization.”	Pamoja staff, health care providers, and local chiefs and assistant chiefs recognized the vulnerability of adolescent girls and young women to new HIV infections, acknowledging the necessity of rapid PrEP implementation and scale-up.	–
**Intervention Characteristics:** Aspects of an intervention that may impact implementation success such as relative advantage, complexity		
***Adaptability:*** **“**The degree to which an intervention can be adapted, tailored, refined, or reinvented to meet local needs.”	The implementation of PrEP through the DREAMS Initiative by Pamoja was adapted to increase PrEP accessibility and decrease community stigma surrounding PrEP. Increased accessibility to PrEP was achieved by allowing PrEP refills to take place at the safe space. Decreased community stigma was achieved through hosting PrEP sensitization meetings for parents and male sexual partners.	–
***Complexity:*** Perceived difficulty of implementation, reflected by duration, scope**,** disruptiveness, and intricacy and number of steps required to implement.	–	The side effects of PrEP was a barrier to implementation, medication uptake and persistence. Some of the adolescent girls and young women reported poor appetite, dizziness, nausea, vomiting, and stomachaches as reasons why their peers have defaulted from PrEP or decided not to be initiated on the medication.
**Characteristics of Individuals:** Individuals’ beliefs, knowledge, self-efficacy, and personal attributes that may affect implementation of people implementing or receiving the intervention.		
***Knowledge and Beliefs about the Intervention:*** Attitudes towards and value placed on the intervention as well as familiarity with facts, truths, and principles related to the intervention	Adolescent girls and young women and PrEP implementers, such as Pamoja staff members and health care providers, recognized the value of PrEP, acknowledging that adolescent girls and young women were at increased risk of new HIV infections. They referenced many risk factors such as poverty, multiple sexual partners, wife inheritance, and boda boda drivers.	–
**Outer Setting:** External influences on intervention implementation including patient needs and resources, external policies and incentives, community culture and attitudes.		
***Patient needs and resources:*** “The extent to which patient needs, as well as barriers and facilitators to meet those needs, are accurately known and prioritized by the organization.”	Through the utilization of the safe spaces, the use of peer mentors and close communication with local community chiefs and assistant chiefs, Pamoja has been able to better understand the challenges to PrEP implementation in addition to the barriers and facilitators to PrEP initiation and persistence among adolescent girls and young women.	Community stigma against PrEP and the frequent relocation of adolescent girls and young women away from DREAMS associated areas continues to remain a barrier to medication uptake and persistence.
	Continuous sensitization meeting surrounding the benefits of PrEP, cultivating positive attitudes around the PrEP use and HIV risk factor reduction among the adolescent girls and young women proved to be a facilitator to medication initiation and persistence.	The health care providers mentioned limited financial and humans resources as barriers to PrEP implementation. They mentioned limited resources in clinics to provide proper testing and follow-up screening for patients on PrEP and the inability to attend safe space meetings due to staff shortages.
**Process:** Strategies used, the presence of key intervention stakeholders and influencers including opinion leaders, stakeholder engagement, and project champions.		
***Engaging:*** “Attracting and involving appropriate individuals in the implementation and use of the intervention through a combined strategy of social marketing, education, role modeling, training, and other similar activities”	Pamoja engaged MoH health care providers, peer mentors and community chiefs and assistant chiefs to help facilitate PrEP implementation. These individuals helped conduct community sensitization meetings, educational sessions with adolescent girls and young women around PrEP and served as role models for PrEP use.	–
***Reflecting and Evaluating:*** “Quantitative and qualitative feedback about the progress and quality of implementation accompanied with regular personal and team debriefing about progress and experience.”	Through the oversight of the MoH, Pamoja created a database to track the amount of adolescent girls and young women initiated on PrEP and monitor the persistence rates within the PrEP program. This information was used to better understand the areas of strength and the areas in which the PrEP implementation strategy by Pamoja could be improved.	–

## Results

### Facilitators to PrEP implementation, uptake and persistence

Facilitators to PrEP implementation, uptake and persistence among adolescents girls and young women were found across all 5 CFIR domains and included the how PrEP was integrated into the DREAMS Initiative HIV prevention programming conducted at Pamoja (Inner Setting), the use of safe spaces and linkage with the existing health care system (Intervention Characteristics), the use of peer mentors (Intervention Characteristics), the continuous education provided at each site (Intervention Characteristics), the education of parents and male sexual partners surrounding the benefits of PrEP (Intervention Characteristics), beneficiaries disclosure of PrEP use (Characteristics of Individuals), the knowledge of risk factors and positive perception of PrEP (Outer Setting), active stakeholder involvement (Process), and the continuous evaluation of quality and progress of PrEP implementation (Process).

### Inner setting (Pamoja): the organization’s willingness to integrate PrEP into the DREAMS initiative

The recognition of the need to adopt PrEP as an HIV prevention method offered through the DREAMS Initiative and Pamoja’s ability to integrate it into the existing DREAMS programming strategy proved to be a significant facilitator. Pamoja staff members acknowledged that adolescent girls and young women were disproportionately affected by new HIV infections and carried some of the highest risks for disease transmission. As a result, they saw the adoption of PrEP as a key addition to their HIV prevention strategy.

*“Most girls between the age 10 and 24 are very vulnerable [to new HIV infections] and research is showing that, especially from 15 to 19, that is the number of girls that is mostly HIV positive in this time and era.” (Pamoja Staff)*

### Intervention characteristics: the use of safe spaces and linkage with the existing health care system

During the initial DREAMS implementation in 2015, Pamoja established safe spaces and engaged local health care providers and government officials. This multi-layered approach, including the involvement of the Kenya MoH, was the foundation of the success of PrEP. Pamoja staff and health care providers identified the safe space as an important facilitator for PrEP initiation and persistence because of the private and friendly environment for adolescent girls and young women to express themselves without fear of discrimination, shame or stigmatization. Additionally, the utilization of the safe spaces allowed for the decentralization of PrEP initiation and medication refills away from the local community clinics, promoting increased access to PrEP by eliminating distance as a reason for poor persistence and defaulting from PrEP.

*“I think that the safe spaces are a brilliant idea because the girls feel safe in the safe spaces. Like in the facility where they might meet some of their relatives from the village and they will shy off from taking their medication. I think that the safe spaces are a nice place for them.” (Pamoja Staff)*

### Intervention characteristics: the use of peer mentors

As observed by health care providers and Pamoja staff, peer mentors were also essential to the success of PrEP implementation. They kept safe-spaces active, ensured continuous education, and communicated with service providers to ensure beneficiaries received different services at the safe space. Mentors also enhance medication persistence by ensuring that adolescent girls and young women honored their PrEP clinic appointments, tracing defaulters and linking them back to services. We observed that without peer mentors, PrEP enrollment and medication persistence would have been difficult.

*“Mentors are the backbone of the DREAMS program. Without them, I think we would not be anywhere near where we are because they are the people who are on the ground and talk to the ladies, talk to the parents, talk to the healthcare community.. .Without them, there wouldn’t be people to help organize the safe spaces, where we meet.. .. They are the people who pass the messages to the girls, even those who are in school that cannot pick up their drugs. They are the people who tell them. . .reminds them of their TCAs.” (Healthcare Provider)*

### Intervention characteristics: continuous education provided at the safe spaces

Adolescent girls and young women noted that continuous education given in the safe space as a part of the PrEP implementation strategy was beneficial. They remarked on how knowledge surrounding PrEP increased their confidence in remaining on the medication and continuously reinforced their reasoning for taking it. Participants were aware that PrEP was not a lifelong intervention, accepting that it was an effective temporary measure as they worked to reduce their overall HIV risk by engaging in the other DREAMS interventions.

*“Yeah, one leaves because she has seen risks are no more or have reduced. This is because we were taught that PrEP isn’t something you take the whole of your life.” (FGD Participant)*

### Intervention characteristics: the education of male sexual partner(s) and parents on the benefits of PrEP

Male sexual partner(s) and parental education was an essential step to the implementation strategy as it promoted PrEP initiation and persistence. Both key informants and DREAMS beneficiaries highlighted the importance of educating partners and parents, emphasizing how this facilitated understanding and disclosure of medication use.

*“But, we faced this challenge and took initiative of educating the male sexual partners. Now, they also have information about PrEP. They have knowledge about PrEP, so it’s even easier to give information to their wives. If the wife say ‘I’m now using PrEP’ it because the husband already has the information.” (Pamoja Staff)*

### Characteristics of individuals: disclosure of PrEP use

Participants, especially PrEP beneficiaries, identified timely disclosure of PrEP use to family members as another facilitating factor for PrEP initiation and persistence. They agreed that disclosure to family members and partners was essential to ensure medication persistence.

*“Disclosing is good.. .there is this time a parent bumped into me along the road and he*

*asked me, ‘the drugs that you wrote for my daughter, PrEP, when is the next refill date*

*because my child is already in school, I thought I would pick it for her and take to her.’ I was surprised because I didn’t know she had already disclosed…so apparently they are free with each other back at home and that made me happy.” (FGD Participant)*

### Outer setting: the knowledge of risk factors among adolescent girls and young women and their positive perception of PrEP

In addition to their awareness of HIV risk factors and knowledge about the purpose and benefits of PrEP, we found that the decision made by adolescent girls and young women to enroll in PrEP was influenced by community, structural and individual related factors. For example, young women said personal experiences with peers and community members determined whether or not one would enroll and stay on PrEP. Participants also identified other cultural and socioeconomic factors (poverty and financial instability, polygamous marriages and wife inheritance, multiple sexual partners, and boda boda drivers) as additional motivators for PrEP initiation and persistence.

*“What do I say? For me the reason why I started using PrEP is that I am in a polygamous marriage and you cannot know how the other person is same to the man, so even if he decides to have as many women as he wants, I am safe.” (FGD Participant)*

Inadequate financial support was identified as a driver for unsafe sexual practices and transactional sex by most adolescent girls and young women. Specifically, financial insecurity was acknowledged by the adolescent girls and young women as one of the reasons why they continue to seek out PrEP while using other DREAMS interventions to reduce their chances of becoming infected with HIV. Some adolescent girls and young women commented on their peers using sex in exchange for commodities and services.

*“But you know the main reason why women get into other relationships outside marriages lack of money. Also, if that’s a school going child, she will get into such relationships because she has needs that aren’t being met. She needs pads, pocket money and yet when she asks the mother, she is told that during her days she used to use blankets as a substitute for pads. So, when she finds one who can do all those things for her, she will definitely be influenced.” (FGD Participant)*

### Process: the engagement of community stakeholders

The Pamoja staff recognized that the utilization of existing community structures and linkages, engaging and collaborating with key opinion leaders and the Kenya MoH were essential to overcoming initial barriers to implementation and ensuring a more successful PrEP roll-out.

*“Okay, implementing this initiative has not been that easy. As I said earlier, when we started administering PrEP, it wasn’t easy because it was a new thing and no one actually has more information. Now after realizing this, in collaboration with the Ministry of Health, [mentors and community volunteers] are taken through trainings and given education materials for PrEP.. .Working together with the mentors and even involving community volunteers, [we] educated girls and even sensitize the community itself on PrEP. .. So with [that] information it was now being taken to the safe spaces with the health care provider, field officer, and the mentor to just educate girls on what PrEP entails.” (Pamoja Staff)*

### Process: continuous evaluation of PrEP implementation and program progress

The implementation of the biomedical services offered by Pamoja (PrEP, HIV testing, contraceptives, post violence care) are also done in collaboration with the Kenya MoH. Without the assistance of the Kenya MoH with continuous data tracking, consistent provision of PrEP, the roll-out would not have been as successful. It allowed for Pamoja and other key implementers to track the progress of PrEP implementation and identify DREAMS beneficiaries would benefit from this intervention.

*“We report every month to [a donor agency] and also to the Ministry of Health. Yeah, because they want to see the trend of PrEP and how it is going up or going down. But at the moment it is going up, which is a good thing. We still need more girls to embrace PrEP because in DREAMS, [PrEP is] our main objective is prevention.” (Pamoja Staff)*

### Barriers to PrEP uptake and persistence

We identified several barriers to PrEP uptake and persistence through Pamoja. These barriers included reported drug side effects (Intervention Characteristics), community stigma against PrEP (Outer Setting), frequent relocation of adolescent girls and young women (Outer Setting), and the limited financial and human resources in the health system to meet the growing demands of PrEP (Outer Setting).

### Intervention characteristics: reported drug side effects

Known side effects of the medication remains a concern among the beneficiaries. Although most participants reported good persistence, some worried about the resulting side effects of the drug. Some complained about poor appetite, dizziness, nausea, vomiting, and stomachaches. These beliefs surrounding drug side effects contributed poor persistence to PrEP and led to defaulting from the PrEP program.

*“What I would say about challenges is the side effects. As one of the people who are on PrEP, the challenge that comes with it is the side effects-because after taking this drug, you may feel sickly in the morning hours and that would make a parent be concerned and want to know why that’s the case….” (FGD Participant)*

### Outer setting: community stigma against PrEP

Despite continued community engagement and education, stigma remains a barrier to successful PrEP implementation among adolescent girls and young women. Most beneficiaries reported that the stigma against anti-retroviral therapy (ART) in the community is transferred onto PrEP because of similar pill appearance and packaging. Respondents also noted that PrEP use is associated with increased promiscuity, commercial sex workers, and people who are infected with HIV.

*“Some of my peers say that I’m a prostitute though for me I know I’m not. I use PrEP so that I prevent HIV infection while others say I pretend to be taking PrEP while I am on ARVs even so this didn’t worry me because I never used to ask anyone, so no one knows my thoughts.” (FGD Participant)*

### Outer setting: frequent relocation of PrEP beneficiaries

Service providers observed that the frequency of relocation among adolescent girls and young women due to schooling or marriage was a barrier to PrEP persistence. Adolescent girls and young women who relocated were more vulnerable to losing consistent access to PrEP because of the loss of connection with Pamoja, leading to defaulting. Most beneficiaries vocalized the same thoughts and admitted that on some occasions, these relocations happened without Pamoja staff members or health care providers knowing.

*“What I think that can make someone to stop using PrEP, is when a woman changes residence, especially in places where access to hospital and PrEP in particular may be a challenge, then I can stop it because returning here frequently will be difficult. It will force me to withdraw because in my new location there is no way out and here, I may not return faster to get PrEP.” (FGD Participant)*

### Outer setting: limited financial and human resources for health care workers to provide PrEP at the health centers and at Pamoja

The insufficient number of health care providers qualified to offer PrEP continues to be a challenge in the current PrEP implementation strategy, making PrEP initiation and persistence more difficult. Barriers to providing PrEP to adolescent girls and young women included low clinic staffing and lack of transport available to health care providers to reach the safe spaces and community events.

*“Staffing is an issue ‘cause at times you like want to you, but you are alone here [at clinic]. So, you are left with your hands tied. So, as much as you really want to go to that baraza or chief camp to maybe enlighten them on PrEP, you find yourself here at the facility. So maybe I can say staffing issue is a challenge.” (Healthcare Provider)*

Limited financial resources at health care facilities has also made the initial initiation and clinical monitoring of PrEP and its side effects difficult. This included either inability or delays in required laboratory tests, resulting in skipping the tests to avoid a delay in PrEP initiation.

*“There are times where funds are limited and to order pertinent lab tests such as liver function tests and creatinine clearance. This is a detriment to the delivery of PrEP, because the inability to adequately screen for health problems or detect potential complications caused by PrEP may delay the initiation and continued administration of the drug to certain individuals.” (Healthcare Provider)*

## Discussion

Through focus groups and interviews with implementers, peer mentors, and adolescent girls and young women we identified key facilitators and barriers to successful PrEP implementation and PrEP initiation and persistence among adolescents girls and young women that spanned multiple CFIR domains and constructs. Facilitating factors included successful integration of PrEP into the DREAMS Initiative by Pamoja, the use of safe spaces and peer mentors, the development of PrEP education sessions for male sexual partners and parents, timely disclosure of PrEP use by beneficiaries, the knowledge of HIV risk factors and positive attitudes surrounding PrEP, the consistent data tracking for PrEP enrollment and identification of high risk individuals, and the collaborative partnerships between Pamoja with its associated partners. We identified drug side effects, PrEP associated stigma, relocation of PrEP beneficiaries, and limited financial and human resources to accommodate increased demand in PrEP as barriers to PrEP implementation.

The capacity for Pamoja to adopt the DREAMS Initiative and its ability to integrate PrEP into its existing HIV prevention programming was key to successful implementation. It’s mission as an organization is to empower communities to identify and address the most significant needs affecting their well-being [22]. The integration of PrEP into its current HIV prevention programming is in line with its goals as an organization, as the prevalence and incidence of HIV infections greatly effects the well-being of the adolescent girls and young women in their community. Community-based organizations, such as Pamoja, are successful in community-based implementation strategies, particularly those pertaining to PrEP. This is because of their ability to establish meaningful ties with marginalized and difficult to reach populations, identify high risk individuals, educate those individuals about PrEP and effectively link them to care [12]. The utilization of community-based organizations will be necessary for continued success in PrEP implementation.

It is well known that the safe space model has been incredibly useful in promoting community-based initiatives. They build social capital and have been shown to increase agency among adolescent girls and young women [8]. The establishment of secure safe spaces for the adolescent girls and young women by Pamoja facilitated PrEP implementation, initiation, and persistence. The positive culture cultivated within safe spaces promotes PrEP initiation and better persistence by encouraging adolescent girls and young women to reduce their HIV risk factors and reminding them to routinely re-evaluate their risk-benefit of taking PrEP. The benefits of the safe space expand beyond continuous sensitization and mobilization of adolescents girls and young women to initiate on PrEP. Safe spaces also serve as key centers to expand accessibility to PrEP among DREAMS beneficiaries in the community, outside of county hospitals and clinics. This strategy provided a more woman-centered approach to PrEP delivery and eliminated many of the barriers associated with time and distance of drug procurement.

Expanding on many existing studies, we also identified peer mentors as key components to successful PrEP implementation. Participants reported that in addition to safe spaces, peer mentors were important to effectively boosting PrEP initiation, as well as persistence, with close follow up and support. These findings are similar to the role of peer mentors in HIV care and treatment programs and is now being adopted into PrEP initiatives [23]. The PrEP Chicago study demonstrates how the formation of social networks and peer mentors have a positive impact on promoting PrEP uptake and persistence in addition to empowering others to take charge of their sexual health [23]. The building of these social connections allows for the filling of informational gaps left by health care providers, increasing trust of PrEP and reducing PrEP related stigma. Reflecting the importance of the mentors for supporting PrEP initiation in Pamoja, PrEP programs will need to determine how to continue peer support for medication persistence as well as ongoing risk reduction even after the PrEP beneficiaries age out and graduate from the program.

We found that the knowledge of HIV risk factors and the positive attitudes that adolescent girls and young women had surrounding PrEP were facilitators to uptake and persistence. Understanding the benefits of PrEP empowered the adolescent girls and young women to use PrEP as an HIV prevention method and it also facilitated disclosure of their PrEP use to their parents and male sexual partners, promoting PrEP initiation and persistence. This is consistent with the existing literature assessing the importance of disclosure on persistence in regard to PrEP. Although it can initially lead to stigmatizing experiences, disclosure to male sexual partners and parents has been described as an empowering way to combat community stigma against PrEP [24, 25]. Additionally, disclosure has been found to improve a young woman’s ability to take the medication and encourage at risk peers to initiate preventative treatment as well.

Unlike previous investigations, our study found the importance of PrEP education sessions for male sexual partners and parents of at-risk adolescent girls and young women, rather than just focusing on the target population. We discovered that this facilitated PrEP uptake and decreased reported PrEP associated stigma. No studies have been performed assessing the overall benefit of PrEP education sessions for the individuals who are closely connected to adolescent girls and young women and can be highly influential to their continuation of PrEP treatment. Velloza et al., further reported that PrEP programs, such as the DREAMS Initiative, can foster community and clinic-based discussion, adherence clubs and normalizing PrEP use [26]. Further exploration of educational programs targeting these individuals could potentially highlight how male sexual partner and parent involvement can reduce PrEP associated stigma.

A critical component to the successful implementation of PrEP Pamoja was its ability to leverage existing partnerships with key local community, health sector and government entities. This was similar to some of the findings published by Chimbindi et al. and Djomand et al. [11, 13, 27]. These studies showed that multi-sectoral collaborations in addition to strengthening existing resources and policies promoted the rapid expansion of the DREAMS Initiative, contributing to increased PrEP uptake [11, 27]. Djomand et al. specifically emphasized the critical importance of stakeholder meetings with strong ongoing engagement from the MoH, the government’s active ownership over its national PrEP program, and the promotion of PrEP outside of the clinical setting to achieve successful scale-up of PrEP services [13].

We found the process of monitoring of PrEP implementation within the context of the DREAMS Initiative as an additional facilitating factor to PrEP implementation. Pamoja has adopted a strong data collection protocol and compiles it into a country wide monitoring and evaluation database, which is reviewed by the KenyaMoH. The development of these databases has allowed for a more granular analysis of PrEP initiation and persistence across all DREAMS affiliated sites and can better assess PrEP success. The value of having a monitoring and evaluation database for DREAMS program tracking has been described previously, but in our study, the importance was significant as it allowed Pamoja to recognize that it was not capturing all adolescent girls and young women who might be eligible for PrEP in their catchment area [11].

Inadequate clinical resources and staffing was a barrier to PrEP implementation. In an environment that already suffers from staff shortages and limited clinic equipment, the incorporation of PrEP as an additional clinic service, which included the important outreach work, was reported as a challenge by study participants. These providers, who are already responsible for HIV care and treatment, are some of the most over-burdened workers in the Kenyan healthcare system and their growing clinic responsibilities in combination with limited additional support has put strain on the scale-up of PrEP [28]. Our interviews revealed that a large number of the defaulters in the PrEP program was due in part to the shortage of personnel qualified to administer PrEP and inadequate clinical resources for routine screening and monitoring. These barriers are similar to those that came about with early implementation of anti-retroviral therapy (ART), which were resolved by calculating the staffing needs to ART provision and creating a more efficient workflow environment in the clinics, creative task-sharing and decentralization [29–31]. A similar multi-strategy approach will need to be taken when to support effective PrEP scale up, requiring increased resource allocation from the MoH.

An additional challenge to successful PrEP implementation was the relocation of PrEP beneficiaries to areas without access to the DREAMS Initiative or PrEP. Relocation has been described as a challenge in a PrEP clinical trial, but it has not been commonly identified as a challenge to PrEP program implementation [32]. Better strategies are needed to support adolescent girls and young women as they relocate, so they can continue to have access to both PrEP and maintain persistence.

Not surprisingly, PrEP stigma, the existing poverty and financial insecurity of the adolescent girls and young women and community members living in Seme sub-county were also identified barriers. Despite the work to reduce this barrier by Pamoja, some PrEP stigma remained, reflecting the association of the medication with HIV infections and increased promiscuity [33, 34]. Published literature has sought out to qualify the type of stigma surrounding PrEP and devise strategies to counteract it. Continued education at the individual and community level and positive marketing about PrEP have been the most effective strategies in stigma reduction so far, a number of which are already being used by [33, 35] .

The strengths of our study include our comprehensive sample size of participants from different aspects of the DREAMS program and the PrEP implementation strategy, including adolescents and girls and young women taking PrEP. Our study had a number of limitations. We only interviewed young women on PrEP and therefore did not have the opportunity to explore barriers for women who did not choose to start or stopped PrEP. Having this information could have given us more insight about the barriers preventing PrEP uptake among adolescent girls and young women. We did, however, ask key informants and focus group discussion participants about potential reasons why adolescent girls and young women decided not to start PrEP, which helped identify some barriers. Additionally, we did not speak to any health care workers who refused to administer PrEP. This could have uncovered health care worker stigma and other provider related biases influencing PrEP uptake. In addition, as seen in qualitative work, responses could have been biased due to social desirability, which we worked to limit by training the interviewers to encourage exploration of barriers as well as facilitators. Although we analyzes responses based on respondent type (Pamoja staff, health care provider, chief/assistant chief, peer mentor, and adolescent girls and young woman), we did not collect demographic information regarding the participants, so we could not further assess the potential influence of these factors. We also did not record the frequency of the emergent themes during our analysis, so we were unable to provide a quantitative description of our results which could have helped us identify which were more common facilitators and barriers. Additionally, since our study was strictly qualitative, we were unable to quantitatively assess the rates of DREAMS beneficiaries who enrolled and subsequently stopped PrEP, although this is work which is ongoing. Finally, only individuals willing to participate were included which could also have introduced bias, a limitation which is inevitable in research.

## Conclusion

Facilitators and barriers to PrEP implementation spanned multiple CFIR domains and constructs. Identified facilitators promoted successful implementation of PrEP within Pamoja, and increased PrEP initiation and medication persistence among adolescent girls and young women. As important as the facilitators are to the ongoing success of PrEP implementation among this group, it is necessary to address the identified barriers as adolescent girls and young women remain at high risk for newly acquired HIV infections. The continued implementation and successful scale-up of PrEP services is critical to reverse the trends of ongoing HIV transmission among adolescent girls and young women in Kenya and other populations in similar settings.

## Data Availability

The datasets used and/or analyzed during the current study are available from the corresponding author on reasonable request. Please contact the corresponding author, Maya Jackson-Gibson (maya.jackson-gibson@northwestern.edu), for any requests for data access.

## Abbreviations

**ART**: Anti-retroviral therapy
**CBO**: Community Based Organization
**CFIR**: The Consolidated Framework for Implementation Research
**DREAMS**: Determined Resilient, Empowered, AIDS-free, Mentored, and Safe women
**FGD**: Focus group discussions
**HIV**: Human immunodeficiency virus
**KII**: Key informant interviews
**MoH**: Ministry of Health
**PEPFAR**: President’s Emergency Fund for AIDS Relief
**PrEP**: HIV Pre-exposure Prophylaxis
